# A 41-Year-Old Man with Two Types of Metachronous Peptic Ulcer Complication due to Zollinger-Ellison Syndrome—Regression of Pancreatic Primary after Chemoembolization of Hepatic Metastases: A Case Report

**DOI:** 10.1155/2011/156937

**Published:** 2011-07-31

**Authors:** Seyed Saeid Sarkeshikian, Mohammad Reza Ghadir

**Affiliations:** Gastroenterology Division, Shahid Beheshti Hospital, School of Medicine, Qom Medical University, Qom, Iran

## Abstract

*Introduction*. Gastrinoma should be suspected when the peptic ulcer(s) is postbulbar, multiple, refractory, or recurrent, or ulcer is associated with nephrolithiasis, hypocalcaemia, or erosive esophagitis. The majority of gastrinomas are malignant. *Case Presentation*. The patient is a 41-year-old Iranian man who has been in good health until 36 months ago when duodenal perforation and two bouts of upper GI bleeding (GIB), each two months apart occurred. He also mentioned mild watery diarrhoea and decreased appetite. Serum gastrin level was elevated. Abdominal CT scan revealed pancreatic mass and three enhancing hepatic masses. CT-guided pancreatic biopsy revealed monotonous cells. Chemoembolization of hepatic metastases was done. New ct images 6 months later showed nearly total regressed hepatic and pancreatic lesions. *Conclusion*. Beside previously defined situations that take gastrinoma into account as the etiology of PUD, accumulation of PUD complications is highly suggestive of Zollinger-Ellisone syndrome (ZES). Regression of pancreatic primary after chemoembolization of hepatic metastases is unexplainable at the present time.

## 1. Introduction

Peptic ulcer disease (PUD), specially duodenal ulcer, is a chronic disease if it's two main causes, *Helicobacter pylori * infection (*H. pylori*) and nonsteroidal anti-inflammatory drugs use, have not been addressed.

 Gastrointestinal bleeding (GIB), perforation, and gastric outlet obstruction are the complications of PUD.

 Upper gastrointestinal (UGI) bleeding secondary to peptic ulcer is a common medical condition that results in high patient morbidity, although it appears to be becoming a less common cause of UGI bleeding.

 Nowadays with more and more improvement of sanitation and *H. pylori* eradication the rate of idiopathic ulcers are increasing. Perforation, another complication of duodenal ulcer, involves the anterior wall of the duodenal bulb in most cases. Perforated gastric ulcers usually involve the lesser curvature [1, 2]. Hypergastrinemia and its pancreatic variant, Zollinger-Ellisone syndrome (ZES), should be borne in mind in the presence of complications, especially if nonsteroidal anti-inflammatory drugs and *H. pylori* are not the causes or diarrhea is also present [3]. Diarrhea is the only symptom in ten percent of patients [4] and appears to be less frequent in ZES patients with MEN I syndrome than in sporadic ZES [5]. After insulinoma, gastrinoma is the commonest islet cell tumor [6]. However, unlike insulinomas, the majority of gastrinomas are malignant. An equal number of gastrinomas occur in the pancreas and duodenum. The majority of duodenal gastrinomas are small (<2 cm in size), but the majority of the pancreatic and peripancreatic tumors are greater than two cm in size. The larger the gastrinoma is, the more likely there will be metachronous liver metastases [7]. Gastrinomas metastasize to the liver, lymph nodes, and bone and rarely elsewhere, such as to the heart [8]. MEN I syndrome, an autosomal dominant disease which beside pancreas (gastrinoma, or other islet cell tumors) involves parathyroid and pituitary glands is present in nearly one third of ZES patients [9]. There is a slight male preponderance with a mean age of 41 years and a mean delay in diagnosis of five years. Beside initial correction of hypersecretion state with potent proton pump inhibitors (PPI), surgery for cure intention in nonmetastatic sporadic disease is the optimal choice. In the hands of an experienced surgeon, up to 50 percent of these patients will be cured [10]. Vagotomy may also be done in the same session and is specially beneficial in uncured patients [11]. The purpose of the case presentation is to introduce a ZES patient with an unusual metachronous occurrence of two types of peptic ulcer complication and also his unexpected regression of primary pancreatic mass after chemoembolization of hepatic metastases.

## 2. Case Presentation

The patient is a 41-year-old Iranian man who has been in good health except mild diarrhea until 36 months ago, when he was suddenly afflicted with severe generalized abdominal pain and rebound in abdominal physical examination. He was attended by surgeon and was operated on. Surgical diagnosis was perforated duodenal ulcer. After discharge, he was prescribed omeprazole for four weeks without any investigation for *H. pylori* infection. He hasnot had any past medical or drug history before operation but he mentioned mild watery diarrhea and decreased appetite without weight loss since a few months ago. One month after termination of omeprazole course, acute upper GI bleeding as melena occurred and he was again admitted in another hospital. Endoscopy was done. A small bulbar ulcer was the cause. Rapid urease test (RUT) was positive. Triple anti-*H. pylori* therapy was completed, and omeprazole was continued for another one month. Twenty days after termination of second course of omeprazole therapy urease breath test was done which was negative for active *H. pylori* infection. No further medication was administered. Approximately ten days later, another bout of upper GI bleeding in the form of melena occurred. He was admitted again in the hospital. Endoscopy revealed duodenal ulcer. RUT was negative. Regarding the history and unusual accumulation of peptic ulcer complications in spite of usual management, hypersecretory states such as gastrinoma were suspected. The result of serologic tests at that time is shown in Table 1. 

**Figure 1:**
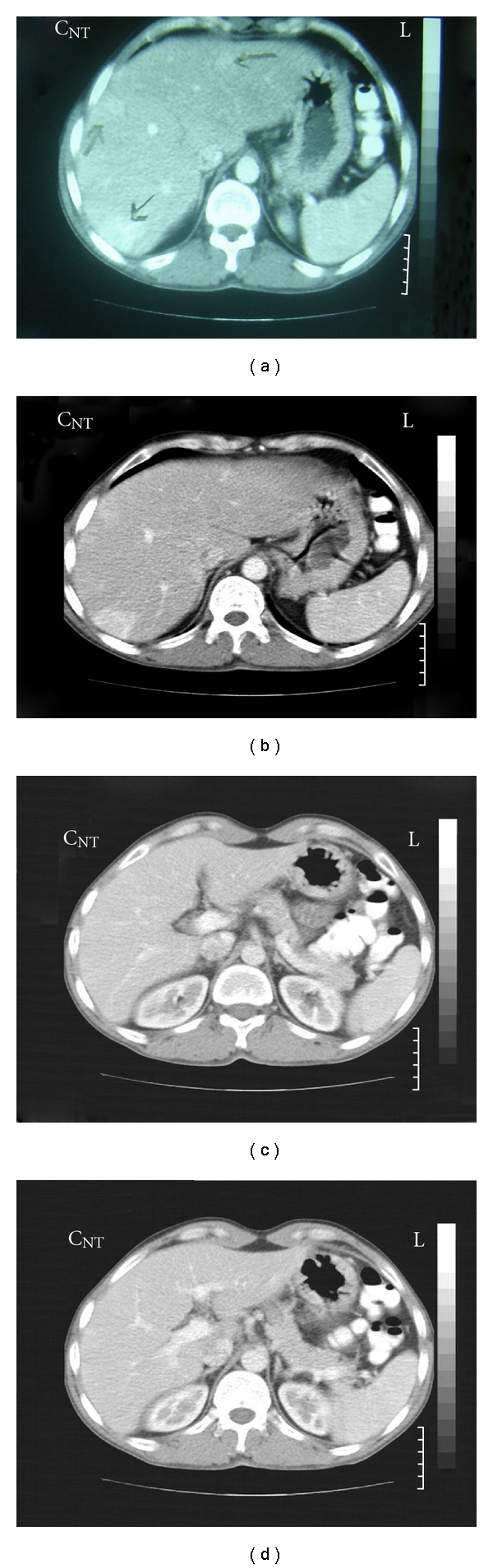
Pancreatic head mass lesion with peripheral enhancement (in favour of an islet cell tumor) and three enhancing lesions in both hepatic lobes, in favour of hypervascular metastasis.

**Figure 2:**
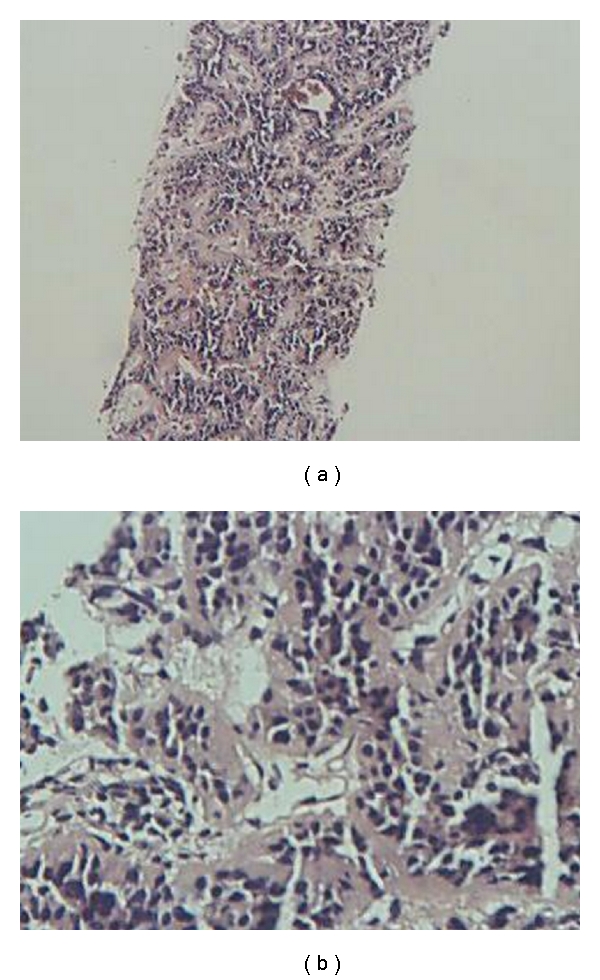
Monotonous cells look like gland islets with preservation of the regular cords, compatible with gastrinoma.

**Figure 3:**
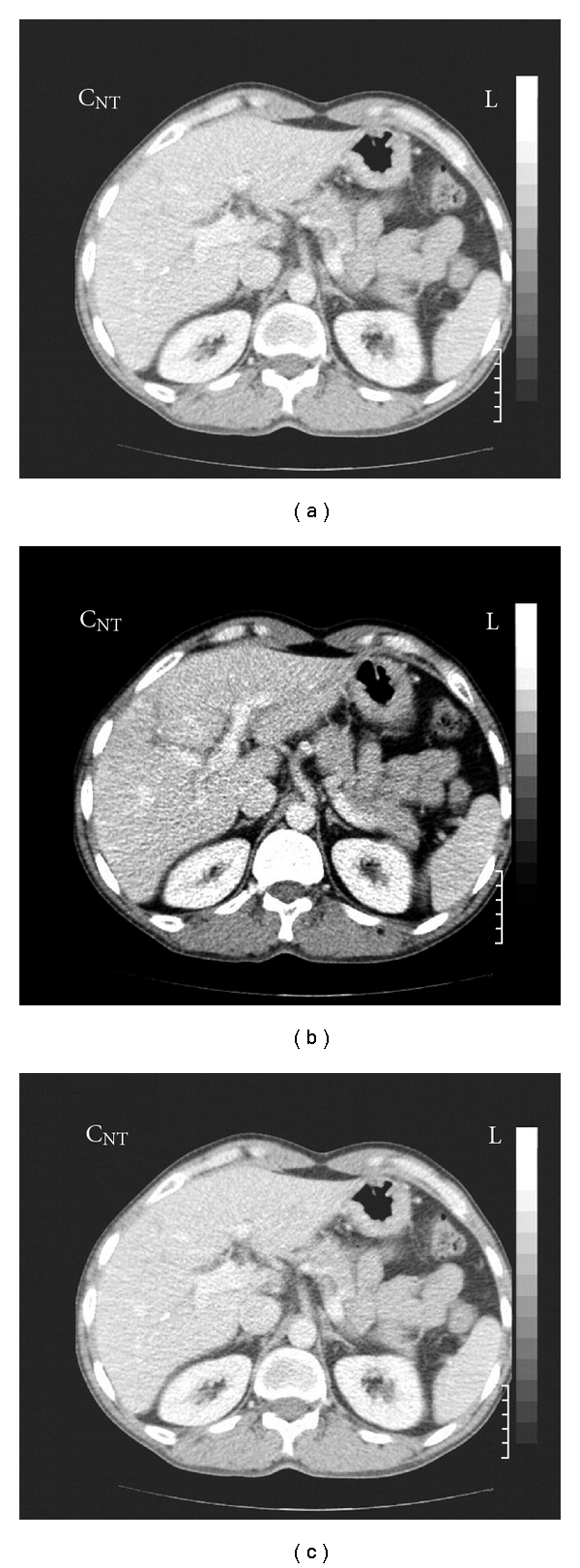
Regression of hepatic lesions 18 months after chemoembolization.

 Abdominal computed tomography (CT) scan revealed a 10 × 8 mm lesion in head of pancreas with peripheral enhancement (in favor of an islet cell tumor) and three superficially located enhancing lesions in both hepatic lobes, in favor of hypervascular metastasis (Figure 1). CT-guided biopsy of pancreatic lesion was done. Pathologic result was as follows: section reveals fragments of tissue including pancreas with a benign neoplasm composed of monotonous cells looking like gland islets with preservation of the regular cords. No any nuclear atypia was seen (compatible with gastrinoma) (Figure 2). Angiography and chemoembolization of hepatic metastatic lesions were done using gel foam, Lipidial, Mitomycin, and Adriablastin. A short while after the procedure, the patient felt severe abdominal pain, that was managed symptomatically with opioid analgesics, and omeprazole 20 mg daily was continued. Now, after 36 months, the patient is in good health and receives omeprazole 20 mg daily. New CT images showed a questionable faint enhanced lesion which shows decreased size and diminished enhancement compared to pre-embolization study. The pancreatic head is prominent without any apparent mass lesion (Figure 3).

## 3. Discussion

Peptic ulcers are used to recuring in similar manner as previous presentations. Metachronous occurrence of two types of complication in a single patient is extremely rare. Persistent *H. pylori* infection, the use of nonsteroidal anti-inflammatory drugs, smoking, and acid hypersecretory states are the causes of ulcer recurrence. Gastrinoma specifically should be suspected if ulcers are postbulbar, multiple, refractory, or recurrent despite *H. pylori* eradication or ulcer is associated with nephrolithiasis, hypercalcemia, or erosive esophagitis or strong positive family history of PUD. Relative to older studies in which multiple ulcers, peptic ulcer complications, and ulcer in atypical locations were frequent, most patients today with ZES have typical duodenal ulcer and up to 29% have no ulcer at diagnosis [12]. In the given patient, decreased appetite, diarrhea, recurrent duodenal ulcer despite *H. pylori* eradication, and the presence of two metachronous PUD complications support the clinical suspicion of ZES. In highly selected patients in whom the perforation seems to have sealed, non operative therapy may be appropriate [13]. In some rare cases, hypergastrinemia due to gastrinoma may be the cause of recurrence or ulcer nonhealing. The liver is the major metastatic site for gastrinomas, as it is with other islet cell tumors. The second most common site is bone (7 percent of patients in one series), almost all of which occur in patients who also have liver metastases [14]. Somatostatin analogs such as octreotide specially in octreoscan positive metastatic tumors can reduce gastrin levels and may slow tumor growth [15]. Hepatic resection may be considered for the treatment of metastatic liver disease in the absence of diffuse bilobar involvement, compromised liver function, or extensive extrahepatic metastases (e.g., pulmonary, peritoneal). Although the majority of cases will not be cured by surgery, prolonged survival is often possible, given the slow-growing nature of these tumors. Hepatic arterial embolization with or without selective hepatic artery infusion of chemotherapy is frequently applied as a palliative technique in patients with symptomatic hepatic metastases who are not candidates for surgical resection. The response rates associated with embolization or chemoembolization, as measured either by decrease in hormonal secretion or by radiographic regression, are generally greater than 50 percent [16]. However, the duration of response can be brief, ranging from 4 to 24 months in uncontrolled series [17, 18]. In one of the largest reports of 81 patients undergoing embolization or chemoembolization for carcinoid tumor, the median duration of response was 17 months, and the probability of progression-free survival (PFS) at one, two, and three years was 75, 35, and 11 percent, respectively [17]. A second series of 69 patients with carcinoid and 54 with pancreatic islet cell tumors suggested better results for carcinoid (response rate 67 versus 35 percent, median PFS 23 versus 16 months, and median overall survival 34 versus 23 months) [18]. The addition of chemotherapy to hepatic artery embolization seemed to benefit islet cell tumors but not carcinoids. Early studies noted a significant incidence of severe postembolization complications, including renal failure, hepatic necrosis, and sepsis. More recently, improvements in technique have reduced the incidence of such complications, making embolization an important and generally safe treatment option for patients with metastatic NETs [17]. Nevertheless, careful patient selection is mandatory because of treatment and disease-related adverse effects, which can range from transient symptoms (pain, nausea, fever, fatigue) as occurred in our patient, biochemical abnormalities (elevated liver enzymes) to florid carcinoid crisis which may be fatal. Regression of pancreatic primary mass after chemoembolization of hepatic metastases was a unique and unexpected event. At the present time, there is not a documented explanation. However, a variant vascular communication between tributaries of hepatic and pancreaticoduodenal arteries or unusual high sensitivity of pancreatic tumoral lesion to chemotherapeutic agents leaked to systemic circulation may be the case [19]. Radiofrequency ablation, cryoablation, and liver transplantation may also be beneficial as liver-directed therapies. Experience with Chemotherapy and targeted radiotherapy for metastatic gastrinoma is limited [20]. In an illustrative series, 185 patients with ZES were followed prospectively for a mean of 12.5 years [21]. The following results were noted: liver metastases were found in 24 percent of patients at the time of diagnosis; the majority of these patients had a primary pancreatic neoplasm, and 67 percent had primary tumours that were greater than 3 cm in size. Patients with liver metastases had a 10-year survival of only 30 percent compared to a 15-year survival of 83 percent in those without liver metastases. Patients with lymph node metastases had the same mortality as those who were free of visceral metastases. Patients with MEN 1 had a significantly lower rate of metastasis at the time of initial diagnosis (6 percent); their high overall survival rate (100 percent at 20 years) reflected this fact.

## Figures and Tables

**Table 1:** Laboratory characteristics of the patient.

	patient	Normal range
Serum gastrin	300 pg/mL	up to 105 pg/mL
Serum calcium	8.8 mg/dL	8.5–10.5 mg/dL
Serum phosphorus	3.4 mg/dL	3–5 mg/dL
Serum alkaline phosphatase	191 u/L	Up to 270 u/L
Serum parathyroid hormone	40 pg/mL	10–65 pg/mL

## Abbreviations

GIB: Gastrointestinal bleeding
PUD: Peptic ulcer disease
UGI: Upper gastrointestinal
PPI: Proton pump inhibitor
MEN: Multiple endocrine neoplasia
ZES: Zollinger Ellisone syndrome
RUT: Rapid urease test
CT: Computed tomography.
